# Novel Combinatorial Approaches to Tackle the Immunosuppressive Microenvironment of Prostate Cancer

**DOI:** 10.3390/cancers13051145

**Published:** 2021-03-08

**Authors:** Erin G. Shackleton, Haleema Yoosuf Ali, Masood Khan, Graham A. Pockley, Stephanie E. McArdle

**Affiliations:** 1 John van Geest Cancer Research Centre, School of Science and Technology, Nottingham Trent University, Nottingham NG11 8NS, UK; erin.shackleton2019@my.ntu.ac.uk (E.G.S.); haleema.yoosufali2019@my.ntu.ac.uk (H.Y.A.); graham.pockley@ntu.ac.uk (G.A.P.); 2 Department of Urology, University Hospitals of Leicester NHS Trust, Leicester LE1 5WW, UK; Masood.Khan@uhl-tr.nhs.uk; 3 Centre for Health, Ageing and Understanding Disease, School of Science and Technology, Nottingham Trent University, Nottingham NG11 8NS, UK

**Keywords:** prostate cancer, PAP, tumour-microenvironment, vaccine, β-blockers, HDACi, MDSC

## Abstract

**Simple Summary:**

The mainstay of treatment for advanced prostate cancer (PCa) is androgen deprivation therapy (ADT), and although patients initially respond, almost all will eventually develop progressive and metastatic disease. Metastatic, hormone-resistant prostate cancer is essentially incurable. A key problem with ADT is that it is not effective in the long term, partly due to the fact that prostate cancers can switch from requiring androgen to grow to surviving without it and these new tumor cells are more sensitive to stress hormones released by the brain as a consequence of chronic stress/depression. Moreover, both extensive treatment with ADT and chronic stress/depression lead to the recruitment of immature myeloid-derived suppressor cells (MDSCs) which are known to suppress immune responses against tumors. The future of prostate cancer treatments will most certainly include a combination of vaccine with beta-blockers (to interfere with signals from stress hormones) and/or the use of histone deacetylase (HDAC) inhibitors which prevent the function/recruitment of MDSC where timing will be of critical importance.

**Abstract:**

Prostate cancer (PCa) is the second-most common cancer in men worldwide and treatment options for patients with advanced or aggressive prostate cancer or recurrent disease continue to be of limited success and are rarely curative. Despite immune checkpoint blockade (ICB) efficacy in some melanoma, lung, kidney and breast cancers, immunotherapy efforts have been remarkably unsuccessful in PCa. One hypothesis behind this lack of efficacy is the generation of a distinctly immunosuppressive prostate tumor microenvironment (TME) by regulatory T cells, MDSCs, and type 2 macrophages which have been implicated in a variety of pathological conditions including solid cancers. In PCa, Tregs and MDSCs are attracted to TME by low-grade chronic inflammatory signals, while tissue-resident type 2 macrophages are induced by cytokines such as IL4, IL10, IL13, transforming growth factor beta (TGFβ) or prostaglandin E2 (PGE2) produced by Th2 cells. These then drive tumor progression, therapy resistance and the generation of castration resistance, ultimately conferring a poor prognosis. The biology of MDSC and Treg is highly complex and the development, proliferation, maturation or function can each be pharmacologically mediated to counteract the immunosuppressive effects of these cells. Herein, we present a critical review of Treg, MDSC and M2 involvement in PCa progression but also investigate a newly recognized type of immune suppression induced by the chronic stimulation of the sympathetic adrenergic signaling pathway and propose targeted strategies to be used in a combinatorial modality with immunotherapy interventions such as ICB, Sipuleucel-T or antitumor vaccines for an enhanced anti-PCa tumor immune response. We conclude that a strategic sequence of therapeutic interventions in combination with additional holistic measures will be necessary to achieve maximum benefit for PCa patients.

## 1. Prostate Cancer Background

Prostate cancer (PCa) is the second-most common cancer in men worldwide, accounting for 13.5% of all new cancer cases in men in 2018 [1]. Incidence rates vary considerably worldwide, with the highest frequencies in Westernized countries such as those in Oceania (79.1 per 100,000 people), America (73.7) and Europe (62.1) while less-developed areas such as Africa and Asia experience a significantly lower incidence, with rates of 26.6 and 11.5, respectively [2]. Interestingly, mortality rates do not align with incidence rates, with elevated mortality rates in Sub-Saharan Africa and the Caribbean. There may be multiple explanations for this global disparity including (i) genetic background, (ii) lifestyle and environmental factors, (iii) proportion of aging populations, (iv) overdiagnosis in well-developed countries, and (v) other factors we are not yet aware of. Despite declining, global mortality rates, the age-adjusted incidence is significantly increasing, although this is at least in part due to increased PCa testing. Following the commercial availability of prostate-specific antigen (PSA) testing in the mid-to-late 1980s, the incidence has dramatically increased in proportion to amount of testing carried out by individual countries, leading to overdiagnosis [3]. Epidemiological overdiagnosis is defined by detection which would otherwise not have been diagnosed within the patient’s lifetime and is a key influencing factor in the disparity between the incidence rates in well-developed and developing countries. Overdiagnosis rates vary widely and range from 1.7% to as high as 67% [4]. Conversely, because disease progression is most often indolent and asymptomatic, latent undiagnosed tumors are found in 36% of autopsied men aged 70–79 [5], with numbers increasing exponentially in later years of life.

For a disease as common as prostate cancer, very little is known about its aetiology and only a few risk factors have been identified. Incidence rates and mortality for PCa also vary greatly between ethnic groups, suggesting ethnic and genetic predisposition. Mortality rates are highest among males of African descent in Sub-Saharan Africa, the Caribbean and the United States [1]. An analysis of biopsy detected PCa in six Sub-Saharan countries found substantially higher Gleason scores than in both African Americans and European Americans, with a score of +8 predominating in Sudan and Uganda [6]. Another study found that Black South African men are at a 2.1-fold and 4.9-fold higher risk of presenting with a Gleason score ≥8 and PSA ≥ 20 ng/mL at diagnosis, respectively, than their African American counterparts [7]. However, there are significant restrictions to producing direct comparisons between Sub-Saharan Africans and African Americans due to differences in the methods of testing, availability of high-quality cancer population data, as well as under-diagnosis and late detection in Africa. Interestingly, a recent study linked chromosomal loci from the KhoeSan ancestry to an increased presentation of high-risk prostate cancer, which may partially explain the increased incidence in Black South Africans compared with African Americans [8].

Ancestral differences in the PCa incidence in addition to family history demonstrate a genetic contribution to PCa risk and etiology. Twin studies have indicated that PCa is among the most heritable of all common cancers [9]. Thus far, Genome-Wide Association Studies have identified 147 loci that account for 28.4% of familial risk, most of which occur commonly but are of low penetrance [10]. Some loci are clinically relevant to the etiology of prostate cancer due to their positioning to nearby oncogenes, DNA damage repair genes, tumor suppressor genes, etc.

Despite the relatively high heritability of PCa, there is overwhelming evidence that lifestyle and environmental factors play a role, though much of the evidence is correlative. Given that the incidence is uniformly significantly higher in developed countries, it has long been suspected that diet and other factors of a “Westernized” lifestyle contribute to increased risk. This is supported by multiple migrant studies where PCa incidence rates increased in Japanese [11], Korean [12] and Chinese [13], Vietnamese [14] migrants after moving to North America. Additionally, there is strong evidence that obesity, which is highly prevalent in Westernized countries, increases the risk of aggressive prostate cancer [15,16]. However, a recent systematic review and meta-analysis suggests that this correlation may be due to the inverse relationship that exists between BMI and PSA levels, resulting in late diagnosis in obese men [17]. While little evidence supports the notion that a high-fat diet increases prostate cancer risk [18], a high-fat diet may influence disease progression and mortality through interleukin-6 (IL-6)-mediated intratumoral infiltration of MDSCs [19]. Interestingly, adult height has been found to have a direct impact on PCa risk, which may be due to polygenic interactions between genes involved in growth, such as IGF-1 [20]. The link between PCa and IGF-1 has also been made in the dietary setting, where a systematic review of 172 studies found that milk consumption conferred increased PCa risk, probably through IGF-1 signaling pathways [21]. This leads to the hypothesis of an intimate interaction between host IGF-1 genes, dietary IGF-1 and growth signaling pathways that ultimately influences adult height and PCa risk. Finally, while it has not been found to be associated with PCa incidence, there is a small but modest association between smoking and PCa death [22].

### 1.1. Diagnosis

Men who present with symptoms such as urination difficulty and/or a family history of PCa are referred to PSA testing and/or digital rectal examination (DRE). PSA is a glycoprotein secreted exclusively by the epithelial cells lining the acini and ducts of the prostate gland that functions to promote sperm motility and dissolve cervical mucus [23]. It is physiologically present in serum at low concentrations (0–4 ng/mL), but can become elevated through prostatic irritation, prostate infection, benign prostatic hyperplasia or the development of PCa and is used as a biomarker for PCa risk and disease progression. While a useful diagnostic and prognostic aid, PSA levels cannot be considered without supporting clinical evidence; for example, 2% of PCa patients harbor an aggressive form of prostate cancer (Gleason score ≥ 7) despite PSA levels < 4 ng/mL [24], and false positives are also common. The DRE is a simple, yet efficient, screening measure whereby the physician feels for palpable nodules, asymmetry or diffuse firmness on the prostate gland through the rectal passageway. However, this technique similarly lacks accuracy, with a reported positive predictive value to be between 5 to 30% [25]. Where available, the first line of investigation for clinically suspected localized PCa is multiparametric MRI (mpMRI), which is a non-invasive imaging technique that has been successful in more accurately characterizing lesions and ruling out non-clinically relevant PCa [26]. The mpMRI is assigned a Likert score from 1 to 5; those with a Likert score of 3 or greater are referred to prostate biopsy. Prostate biopsy is a requirement for diagnosis and is referred to patients with PSA levels repeatedly exceeding 4 ng/mL and/or palpable nodules upon DRE and/or high Likert score on mpMRI and/or clinical suspicion of PCa [27].

Transrectal ultrasound (TRUS)-guided systematic prostate biopsy is the standard of care for PCa diagnosis and is carried out via needle biopsy through the rectal passageway to extract 10–12 samples in a grid-like pattern over the apical and far lateral regions [28]. The TRUS biopsy is the least invasive technique and is widely accessible. However, this approach suffers several drawbacks including higher infection rates, higher false negative rates and underestimation of Gleason grade. Contemporary MRI-guided biopsies offer enhanced detection of clinically important lesions and include MRI-guided (in-bore) biopsy, fusion biopsy and MRI–TRUS fusion biopsy [26]. While less common, transperineal biopsies are considered preferable to TRUS biopsies by many urologists due to more comprehensive sampling, decreased risk of post-operational infection and comparable cancer detection rates [29]. While not yet recommended by the UK’s National Institute for Health and Care Excellence (NICE) guidelines (unless as part of a clinical trial) [27], transperineal biopsies may be useful in cases where prior TRUS biopsy returned negative but there is clinical suspicion of PCa based on other parameters such as PSA, DRE and high Likert score. Indeed, freehand transperineal biopsies have completely replaced transrectal biopsies at one of the UK’s largest hospitals [30] and is likewise being adopted elsewhere around the world. Finally, transurethral biopsy represents the third prostate biopsy type. However, this method has largely been surpassed by other techniques due to poor diagnostic accuracy.

**Figure 1:**
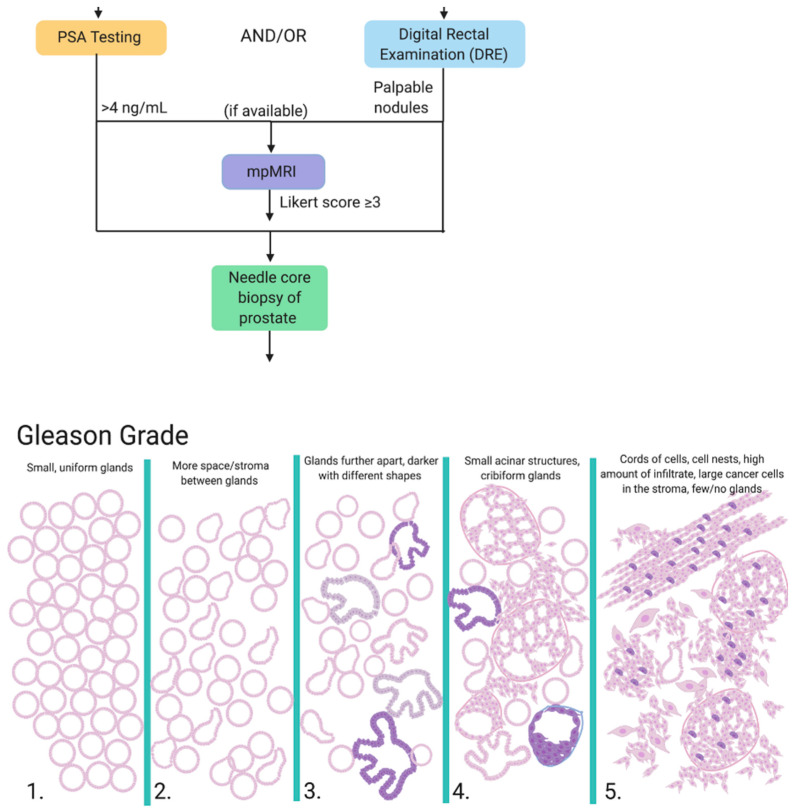
Process of diagnosis and risk stratification for prostate cancer.

Regardless of the biopsy type, samples are given a primary and secondary Gleason Grade from 1 to 5 based on the architecture and state of differentiation of the predominant and second-most prevalent pattern in the sample. These are added together to get the resulting Gleason score, which is assigned a Gleason Grade Group (Figure 1). An important distinction is a 3 + 4 score versus a 4 + 3 score, as patients with a 4 + 3 score present with higher levels of PSA at diagnosis and are at a 3-fold increased risk of metastasis [31]. Additionally, it is notoriously difficult to adequately sample or even properly capture prostate tumors; 21–28% of tumors that are located on the anterior side of the prostate are missed and 14–17% are under-graded through current techniques [32].

### 1.2. Treatment

Given the generally indolent disease progression and hinderance to quality-of-life PCa treatment options impose such as incontinence and impotence, treatment is considered in the context of age and risk group. Approximately 45% of tumors will not progress in the patient’s lifetime and are deferred to ‘Active Surveillance’ or ‘Watchful Waiting’, both of which aim to minimize treatment-related toxicity [33]. It has been established in multiple randomized clinical trials that radical prostatectomy does not significantly reduce prostate cancer mortality in those with localized PCa [34,35], and the risk of death from alternative causes supersedes the risk of death by prostate cancer itself, particularly in men over 60 years old. There has been an increasing role for patient preferences in treatment decision making, as similar health outcomes are achieved through shared decision making [36]. Some of the main concerns leading to patients preferring deferred management over treatment options such as prostatectomy and radiation therapy is the reduced quality of life associated with these treatments including incontinence and erectile dysfunction, which are reported to occur in approximately 20% and 70% of patients, respectively [37].

Beyond expectant management, patients with localized PCa have two primary options: radical prostatectomy (RP) or radiation therapy. RP may be conducted through laparoscopic or robot-assisted (RARP) techniques and may be performed within as little as 2 weeks from the time of biopsy [38]. A recent systematic review and meta-analysis of two randomized controlled trials and 9 prospective studies indicated no significant difference between the open, laparoscopic or RARP techniques in terms of complications, biochemical reoccurrence, urinary continence and erectile function, although RARP resulted in significantly lower blood loss [39]. The type of external beam radiation used for the treatment of PCa is intensity-modulated radiotherapy (IMRT), which may also be image-guided (IGRT) [27]. Less commonly, low-dose (permanent implantation) and high-dose (temporary implantation) brachytherapy may be used in conjunction with IMRT/IGRT on patients with intermediate- and high-risk localized PCa who satisfy certain conditions [27]. Other still-experimental treatment modalities include cryosurgery [40], high-intensity focal ultrasound [41], irreversible electroporation [42] and photodynamic therapy [43]. However, these are not recommended unless part of a clinical trial. Once diagnosed and a baseline level is established, PSA testing represents an essential monitoring measure for detection of local reoccurrence and metastatic disease, although it is not possible to differentiate between these two scenarios from a PSA test alone. A definitive diagnosis of biochemical reoccurrence is determined through rising PSA levels, radiology and clinical signs of deterioration.

**Figure 2:**
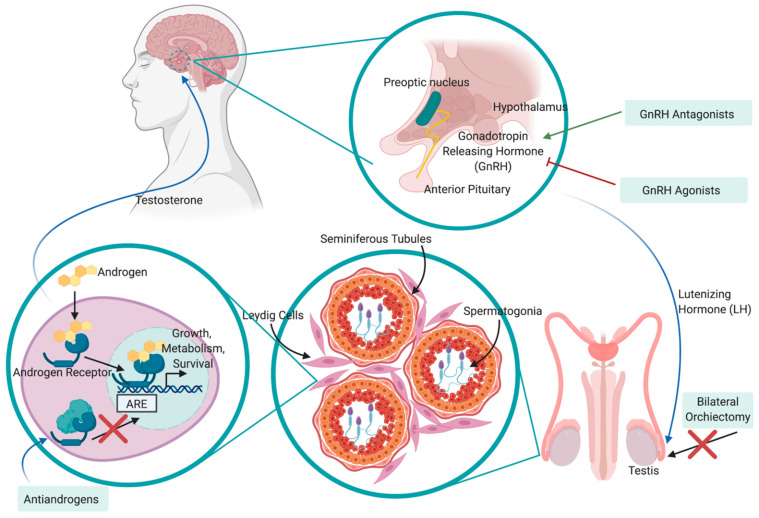
Mechanism of action and feedback loop of ADT on the hypothalamic–pituitary–gonadal (HPG) axis in the treatment of prostate cancer. Luteinizing hormone-releasing hormone (LHRH) is released from the preoptic nucleus of the hypothalamus to induce the secretion of luteinizing hormone (LH) from the anterior pituitary gland. LH moves through peripheral circulation to act on the Leydig cells of the testis, inducing release of testosterone. Testosterone promotes Pca progression by binding to androgen receptors (Ars), translocating to the nucleus, binding to androgen-responsive elements (AREs) and promoting expression of proteins involved in growth, metabolism and survival. This pathway may be disrupted by physical castration (bilateral orchiectomy) or chemical castration via LHRH agonists/antagonists, antiandrogens and CYP17 inhibitors.

ADT remains the mainstay treatment for high-risk and recurrent patients and involves various interferences of androgen hormones or their receptors. ADT was pioneered in 1941 by Huggins and Hodges, who observed that ablation of androgen hormones, either through chemical or physical castration, resulted in the inhibition of tumor growth and cancer-related symptom relief [44]. In the decades that followed, various agents were developed to interfere with the hypothalamus–pituitary–gonadal axis and functioning of the androgen receptor (AR) signaling pathway including anti-androgens, luteinizing hormone-releasing hormone (LHRH) agonists and LHRH antagonists [45] (Figure 2). Anti-androgens work by inhibiting the interaction between dihydrotestosterone (DHT; a derivative of testosterone that is formed in the prostate gland) and the AR; one such antiandrogen is enzalutamide. As a monotherapy, anti-androgens are less effective than bilateral orchiectomy or other chemical forms of ADT in patients due to the fact that they do not reduce serum testosterone levels and are typically used in conjunction with LHRH agonists/antagonists to achieve “complete androgen blockade” (CAB). LHRH agonists stimulate the LHRH receptor, resulting in a downregulation of the receptor following 2–3 weeks of treatment, whereas LHRH antagonists competitively inhibit the receptor from binding to LHRH. The main difference between the LHRH drugs is that through activating the LHRH receptor and subsequent signaling pathway, LHRH agonists result in a transient surge in luteinizing hormone and testosterone levels, while LHRH antagonists achieve testosterone suppression without this initial surge. Additionally, testosterone production can be successfully inhibited from all sources (testes, adrenal glands, PCa cells) via oral administration of abiraterone acetate, which is an inhibitor of the androgen biosynthesis enzyme CYP17. CYP17 functions by hydroxylating 17-hydroxypregnenolone to produce dehydroepiandrosterone (DHEA), which is subsequently converted to testosterone. By targeting biosynthesis in completely different pathway, abiraterone acetate compliments other forms of ADT to successfully achieve very low testosterone levels with improved outcome. While physical castration via bilateral orchiectomy remains an effective and cost-efficient form of ADT, it is less common due to the invasiveness of surgery and permanent consequences. Regardless of the method used, a testosterone level of <20 ng/dL is desirable to maximize therapeutic outcomes.

While Pca is initially highly responsive to ADT, it almost invariably develops resistance within 2–3 years to become castration-resistant prostate cancer (CRPC, or mCRPC if the cancer has metastasized), where the tumor continues to grow despite an absence of testosterone [46]. CRPC is determined by a continuous increase in PSA levels, the progression of a pre-existing disease or the development of new metastases, despite castration-level concentrations of testosterone. The development of CRPC is due to a variety of molecular mechanisms involving AR reactivation including production of androgens via the adrenal glands and Pca cells, androgen-independent AR activation, gene amplifications and mutations, aberrant co-regulator activates and ligand-independent splice variants. The castration-resistant tumor contains a heterogeneous population of cells consisting of fully androgen-insensitive cells, partially androgen-insensitive cells and androgen-sensitive cells, thus warranting ongoing, effective ADT therapy; however, the proliferation of the castration-insensitive population cannot be prevented. mCRPC confers a poor prognosis, with a median survival of 1.14 years [47] and 17% survival rate over 3.5 years [48]. To treat mCRPC, ADT is combined with the chemotherapeutic agent docetaxel, and failing this the patient may be referred to the taxane cabazitaxel [49] or radiation therapy with radium 223 to target bone metastases [50]. Despite the availability of these new agents and the improved overall survival with sequential use, there is no consensus on proper sequencing and the median overall survival remains poor, ranging from 21 to 29 months [51]. This is at least partially due to the development of resistance and cross-resistance mechanisms; for example, it has been demonstrated that acquired resistance to docetaxel induces cross-resistance to cabazitaxel, and that resistance to enzalutamide induces cross resistance to abiraterone [52].

### 1.3. Immunotherapy

With the lack of curative treatment options for mCRPC, various immunotherapies have been clinically investigated with the goal of initiating a robust antitumor response in vivo. Despite the efficacy of immune checkpoint inhibitors such as ipilimumab (anti-CTLA-4) and nivolumab (anti-PD-1) in a variety of cancers including melanoma [53], lung [54], kidney [55] and breast [56], clinical trials for these drugs in Pca have found them relatively inert or even toxic. In two large, randomized phase III clinical trials, ipilimumab did not cause an increase in overall survival when given either before [57] or after [58] treatment with docetaxel. Another phase I clinical trial assessing the safety and antitumor activity of nivolumab on patients with mCRPC also showed negligible response [59]. For the 2–3% of patients with DNA mismatch repair genes increased mutational burden renders either of these drugs effective; pembrolizumab is already FDA approved and investigations for ipilimumab plus nivolumab [60] and durvalumab (anti-PD-L1) plus Olaparib [61] are currently underway. Most recently a phase II clinical study on pre-treated mCRPC patients found positive antitumor activity through treatment with pembrolizumab with a decrease in tumor size in 29% [62]. However, no control arm was assessed for statistical analysis of this result.

**Figure 3:**
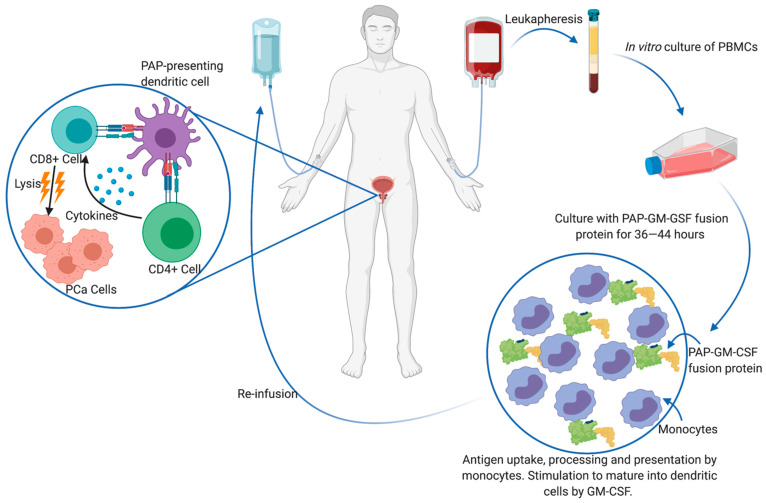
Sipuleucel T mechanism of action.

Although clinical trials for monoclonal antibodies are still ongoing, various antitumor vaccines are currently in the pipeline in the form of antigen-loaded antigen-presenting cells (APCs) [63], peptides [64,65] or loaded DNA vectors [66]. Intratumoral CD8+ T cells have a canonical exhausted phenotype, with a diminished ability to proliferate and produce effector cytokines such as IL-2, IFNg and TNF-a in addition to enhanced expression of immune checkpoint receptors such as PD-1, TIM-3 and LAG-3 [67]. Thus, vaccine approaches aim to present the host immune system with tumor-specific antigens and elicit robust CD8+ T cell activation. Currently, the only FDA-approved immunotherapy for mCRPC is sipuleucel-T (PROVENGE^®^), which is an ex vivo autologous peripheral blood mononuclear cell (PBMC) vaccine [68]. To generate the vaccine, the patient’s PBMCs are isolated through leukapheresis and cultured with the entire prostatic acid phosphatase (PAP) protein conjugated to granulocyte-macrophage colony-stimulating factor (GM-CSF) by a single Gly-Ser linker (PA2024), which is taken up, processed and presented on APCs as PAP epitopes. The GM-CSF conjugate induces maturation of many of these APCs into dendritic cells (DCs) and induces in vitro antigen processing. The entire PMBC cell culture is subsequently re-infused back into the patient, where DCs are thought to activate CD4+ and vaccine-specific CD8+ T cells (Figure 3). PAP is an ideal prostate tumor antigen due to its prostate restricted expression and overexpression that correlates with disease progression [69]. Sipuleucel-T treatment prior to RP has been associated with intratumoral infiltration of CD3+, CD4+, FOXP3- and CD8+ T cells, IFNɣ-detectable responses and Th1-biased activation, which is important for host tumor immunity [70,71]. Additionally, T-cell receptor (TCR) diversity decreases in peripheral blood but increases intratumorally, indicating recruitment of T-specific clones from the peripheral blood to the prostate tumor microenvironment [72]. Sipuleucel-T administration has also been explored in neoadjuvant [70,71] and biochemical recurrent settings [73]. However, it is not FDA approved for these uses.

Interestingly, no study to date has demonstrated solid evidence of PAP only specific CD8+ T cell following Sipuleucel-T treatment. In the landmark study that granted the drug FDA-approval, increased overall survival was achieved (reduced risk of death of 22%, *p* = 0.03), and immune responses against PA2024 were observed via antibody titer ELISA and T-cell proliferation assay [68]. Further phase III trials found cellular and humoral responses against PA2024 and/or PAP [74]. However, these response assays were produced against the entire PA2024 antigen without a GM-CSF control, which begs the question whether Sipuleucel-T’s in vivo effects can be attributed to an adaptive immune response against the fusion protein construct containing GM-CSF rather than the perceived tumor antigen PAP. Indeed, GM-CSF is secreted by activated dendritic cells and exogenous GM-CSF has been suggested to have antitumor activity in patients with advanced prostate cancer [75]. Clinical and pre-clinical research leading up to this study reported T-cell proliferation in response to PA2024 and antibodies against in PA2024 100% of patients, but much weaker and infrequent immune responses were generated against PAP or GM-CSF alone [76,77]. The only report of PA2024-specific CD4+ and CD8+ T cell proliferation and activation is that of Antonarakis et al. [78]. However, this result remains to be externally validated. Although overall survival is significantly improved through administration of Sipuleucel-T, this improvement is limited to an increase of 4.1 months, with no difference in progression-free survival or time-to-clinical progression [68]. From a cost perspective, Sipuleucel-T requires three infusions at two-week intervals for one month and costs a total upwards of £47,000 [27]. The preparation of each dose of Sipuleucel-T is also demanding on pre-immunocompromised patients. A total of 8–14 L of blood (i.e., approximately 1–2 times total blood volume) must be collected, which results in approximately 600 mL of PBMCs to be obtained and reduces the patient’s peripheral WBC blood count by 15–20%. With such a burdensome procedure, low benefit-cost ratio, and unclear capability of CD8+ T cell activation, efforts have been redirected at either improving Sipuleucel-T efficacy or producing an alternative form of immunotherapy.

One reason for the lack of clinical efficacy of immunotherapy in PCa is the low mutational burden of the disease, which results in low mutation-associated neoantigens and low immunogenicity. To improve upon the design of sipuleucel-T and determine the exact immunogenic region of the PAP protein, the John van Geest Cancer Research group identified a 15 mer PAP epitope of amino acids 114–128 that are capable of initiating CD4+ and CD8+ T-cell responses in mice [79]. The identified peptide was selected based on predictive binding to the human leukocyte antigen HLA*A02:01 and 100% homology between the human and murine PAP protein. However, the haplotype HLA*A02:01 represents only ~40% of US Caucasians and ~15% of US African Americans [80], warranting the need for a broader-spectrum peptide. Based on the observation that vaccination with long peptides (20 aa or more) induces more robust immune responses in a wider range of phenotypically diverse individuals [81], the identified peptide was elongated to 42 amino acids and mutated with a Leucine to Alanine substitution at position 14 for additional immunogenicity [82]. As an intermediate-sized peptide, this vaccine meets the requirements of balancing immunogenicity and wide haplotype specificity, with the enhanced ease of purification and lower cost compared to producing a whole protein. In mouse models this immune-vaccine induced robust anti-PAP immunity via MHC-I and delayed tumor growth.

The lack of efficacy of immunotherapy techniques such as immune checkpoint blockade and Sipuleucel-T in prostate cancer indicates other factors at play which result in inhibition of antitumor immunity. One hypothesis for this lack of efficacy is the generation of a potently immunosuppressive tumor microenvironment by regulatory T cells, type 2 macrophages (TAMs) and MDSCs, all of which have been implicated in a variety of solid cancers. The aim of this investigation is to critically assess the role of MDSCs in prostate cancer tumor progression and resistance to immunotherapy, assess methods of targeting them, and propose combinatorial treatment modalities with the ultimate goal of improved immunotherapy efficacy.

## 2. Myeloid-Derived Suppressor Cells

### 2.1. The Tumor Microenvironment

Like all solid tumors, the prostate tumor microenvironment (TME) is a complex and dynamic landscape composed of key tumor-promoting players such as cancer-associated fibroblasts (CAFs), mesenchymal stem cells (MSCs), immune cell infiltrates, vascular and lymphatic vesicles and the extracellular matrix (ECM). Although much has been discovered about the prostate cancer tumor microenvironment, the nature of needle-core prostate biopsies has been a significant obstacle in obtaining a comprehensive understanding, due to inadequate sampling and often incomplete margins.

**Figure 4:**
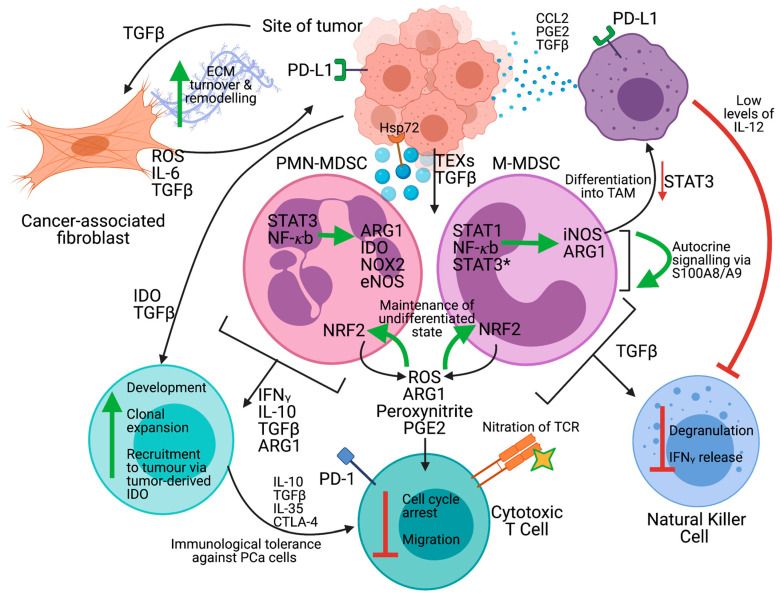
Function of myeloid-derived suppressor cells (MDSCs) within the tumor microenvironment. Square brackets indicate both subsets are involved in the action, asterisk (*) indicates in peripheral circulation only.

CAFs are the most prevalent component of the TME stroma and are responsible for the generation of a ‘reactive stroma’ in response to the presence of PCa cells. This is achieved through accelerated ECM turn-over and remodeling, which permits the release of previously tissue-associated molecules known for promoting growth and survival [83]. This wound-healing response mechanism is continuously maintained by PCa cells, which secrete CAF-promoting TGFβ. In turn, CAFs produce excess amounts of ROS and tenascin-C which facilitate PCa proliferation and migration. MSCs are progenitor cells to CAFs and have been identified in histology specimens from both benign and malignant prostate tumors [84]. MSCs are thought to contribute to tumor progression through pro-angiogenic properties by expressing multiple matrix metalloproteases (MMPs), VEGF and bFGF which degrade the ECM and promote new vessel formation. Additionally, MSCs drive the development and expansion of highly immunosuppressive MDSCs through secreted cytokines and activation of signal transducer and activator of transcription 3 (STAT3) [85,86]. Tumor-associated macrophages (TAMs) are M2-like macrophages that are derived from M-MDSCs which rapidly differentiate in the TME through downregulation of pSTAT3 [87]. TAMs are tumor-promoting as they produce high levels of IL-10 and low levels of IL-12, an essential cytokine for natural killer (NK) cell and TH1 cell activation [88]. In PCa TAMs express CCL22, which potentiates tumor migration and invasion and traffics T regulatory cells (Tregs) to the site of the tumor [89]. Both MDSCs and TAMs induce Tregs, which are known as ‘suppressor T cells’ and whose role is to maintain immunological anergy against self-antigens. The induction of Tregs within the TME, however, dampens antitumor immunity, allowing the tumor to grow uninhibited through expression of various immune checkpoint molecules including CTLA-4, PD-1, LAG-3 and TIM-3 [90]. As Figure 4 demonstrates, MDSCs are central to many of the immunosuppressive responses within the TME. Unfortunately, the extended amount of time from PCa initiation to clinical manifestation (which can be many decades) and the low mutational burden of PCa cells ultimately leads to a tolerized microenvironment with increased numbers of immunosuppressive Tregs, TAMs and MDSCs.

### 2.2. Subpopulations of Myeloid-Derived Suppressor Cells

MDSCs represent a heterogeneous cell population of immature myeloid cells which suppress local T and NK cell activity thereby maintaining immunological anergy [91]. Despite initial reports of the presence of natural suppressor cells in tumor-bearing mice throughout the 1960s and 1970s, MDSC nomenclature and function have only been truly established in the past ~15 years. The accumulation of immature myeloid cells has been reported in almost every type of solid cancer, and the inherent state of chronic intratumoral inflammation both recruits and activates MDSCs through soluble secreted factors. MDSCs arise from a common myeloid progenitor that also produces DCs, monocytes, macrophages and granulocytes [91]. There are two major subpopulations of MDSCs; monocytic (M-MDSC), defined phenotypically in humans as CD33^+^CD11b^+^CD14+CD15^−^CD66^−^ HLA-DR^low/−^ and polymorphonuclear (PMN-MDSC), defined as CD33^+^CD11b^+^CD14^−^CD15^+^CD66^+^HLA-DR^−^. PMN-MDSCs, which bear high morphological and phenotypical similarity to terminally differentiated neutrophils, can be additionally distinguished via LOX-1 expression, which is virtually undetectable in neutrophils [92]. Currently, there are no unique individual markers for MDSCs and therefore demonstration of immunosuppressive function is required for definitive identification.

The two major subpopulations represent two states of differentiation along a common lineage (rather than two separate lineages) and utilize distinct regulatory pathways and mechanisms of immunosuppression. Both subsets rely on the STAT3 signaling pathway functioning, yet only PMN-MDSCs rely on it for immunosuppressive functioning [93]. The main immunosuppressive mechanisms utilized by PMN-MDSCs are STAT3-mediated overexpression of NADPH oxidase (NOX2) and endothelial nitric oxide synthase (eNOS), whereas M-MDSCs upregulate levels of inducible NOS (iNOS), which is mediated by STAT1 and NF-kB [88]. Both subtypes express arginase 1(ARG1) and a variety of pro-inflammatory cytokines to exert immunosuppression. Circulating M-MDSCs maintain high levels of STAT3 signaling until they reach the site of the tumor, where hypoxia induces a rapid downregulation of STAT3 resulting in differentiation into TAMs. The release of ROS by MDSCs is essential for their immunosuppressive function as well as retaining their undifferentiated state. For most cells, persistent oxidative stress induces apoptosis due to the damage of lipids, proteins and carbohydrates; curiously, MDSCs survive despite continued elevated production of ROS. This protection from apoptosis is mediated though upregulation of glycolysis, which results in the antioxidant glycolytic metabolite phosphoenolpyruvate [94], and constituent activation of the endogenous antioxidant-regulator nuclear factor erythroid 2-related factor 2 (NRF2) [95]. Activation of NRF2 releases this transcription factor to translocate to the nucleus, bind to antioxidant response elements (AREs) and regulate the transcription of genes involved in cytoprotective proteins. Therefore, the upregulation of both glycolysis and NRF2 results in additional antioxidants to mitigate rising ROS levels. The production of ROS by MDSCs also maintains them in an undifferentiated state; this has been demonstrated by the fact that abolishing ROS through both the addition of the H_2_O_2_ scavenger catalase and the knockout of NOX2 induces MDSCs to differentiate into macrophages in tumor-bearing mice [96,97].

Determination of which subset is the most (a) prevalent and (b) immunosuppressive in PCa remains controversial, as different reports use different combinations surface markers and obvious differences exist between serum, lymphoid and tumor populations. Multiple sources report M-MDSC as the predominating subset in the serum of treated, untreated and mCRPC patients [98,99,100,101]. This is contested by one study on newly diagnosed PCa patients, which found PMN-MDSCs to be the major subtype in peripheral blood samples [102]. Relatively few studies have assessed MDSC subtype frequencies in prostate tumor tissue, which does not appear to follow the same trends as those found in the periphery. PMN-MDSCs represent the overwhelming majority of MDSCs in both PTEN/SMAD4-deficient mice [103], and in human localized and metastatic prostate cancer tissue [104] and predominates in the stroma, rather than the epithelia, of both primary tumors and metastases [105]. In PCa-harboring lymph nodes of hormone-naïve patients, PMN-MDSCs and STAT3 levels are both significantly elevated compared to the peripheral blood, whereas M-MDSC frequency is similar in both locations [106]. Thus, given the available evidence it therefore seems likely that M-MDSCs predominate in the periphery while PMN-MDSCs represent the majority of intratumoral MDSC populations, which is consistent in lymph nodes following metastasis of the primary tumor. In general, M-MDSCs have been said to be more potent in their immunosuppressive activity on a per-cell basis [107]. However, whether this remains true for PCa and other cancers is also debated. Several studies have indicated PMN-MDSC as the most potent functional subtype in both PCa and other cancer types as determined by T cell proliferation assays in both PCa and non-PCa mouse and human settings. Only PMN-MDSCs, and not M-MDSCs, are capable of blocking IFNg and granzyme B production from CD8+ T cells [104], and this immunosuppressive activity is mirrored by molecular characteristics of PMN-MDSCs such as ARG1, NOS2, and STAT3 levels [108]. Additionally, targeting PMN-MDSC NO pathways though deletion of eNOS and gp91phox (the gene for NOS2) or addition of a PNT scavenger successfully negates the immunosuppressive function of PMN-MDSCs without affecting M-MDSC activity [109]. It is possible that the decreased potency of M-MDSCs is due to the cell type they primarily target. Idorn et al. demonstrated significant iNOS-mediated inhibition of M-MDSCs from PCa patients on CD4+ T cell proliferation and only weak inhibition on CD8+ T cells [100]. The existing evidence therefore suggests that PMN-MDSCs represent the more potent subtype due to its CD8+ T cell-specific mechanism of immunosuppression, whereas M-MDSCs exert immunosuppressive effects primarily on CD4+ T helper cells, which help regulate the CD8+ T cell response.

### 2.3. Expansion and Activation

Under normal conditions, MDSCs respond to myelopoiesis signals or acute inflammation to expand and differentiate into macrophages, DCs, granulocytes, monocytes or activated neutrophils as required [88]. This activated immune response is fundamental in neutralizing foreign invaders, which are recognized through pathogen-associated molecular patterns (PAMPs) or danger-associated molecular patterns (DAMPs), resulting in a robust burst of respiration, release of pro-inflammatory cytokines, and phagocytosis. Accordingly, this lethal cytotoxic activity is terminated upon cessation of the acute inflammatory signal to prevent autoimmune attack. However, under conditions of chronic and relatively low-intensity inflammation such as cancer, modest but persistent myelopoiesis is initiated and MDSCs result as a failure to terminally differentiate, giving rise to a cell population with a potent capacity to suppress T cell activation.

Although MDSC accumulation and activation is a complex phenomenon, these low-intensity inflammatory signals can be generally divided into those responsible for MDSC expansion within the TME, and those responsible for their immunosuppressive activation. Soluble tumor-derived factors responsible for the expansion of MDSCs include granulocyte-macrophage colony-stimulating factor (GM-CSF), granulocyte CSF (G-CSF), macrophage CSF (M-CSF), stem cell factor (SCF), vascular endothelial growth factor (VEGF), polyunsaturated fatty acids and semaphorin 4D [110,111]. Within the TME, there is continuous low-grade secretion of inflammatory cytokines such as IFNg, TNF, IL-1B, IL-4, IL-6, IL-10, IL-12, IL-13 and high mobility group box 1 (HMGB1), among others. This chronic state of inflammation results in pathological MDSC activation, largely through NF-kB, STAT1, STAT3 and STAT6 pathways. Of all the signals, GM-CSF and IL-6 appear to be especially potent in activating MDSCs. Compared to M-CSF and G-CSF, GM-CSF induces both the largest expansion and most functional immunosuppressive MDSCs [112] and GM-CSF and IL-6 are sufficient to induce monocyte differentiation into M-MDSCs [113]. Mechanistically, GM-CSF/IL-6-dependent induction is potentiated by PGE2. MDSCs also maintain an autocrine feedback look that sustains their accumulation and immature state through the secretion of S100A8/A9 proteins; this expression is induced by STAT3 signaling and results in pro-survival NK-kB signaling [114]. In recent years, the tumor-secreted cytokines CXCL6 [103] and IL-8 [115] have been implicated in trafficking of MDSCs to the TME via the receptor CXCR2, which is expressed on MDSCs.

The TME is a hostile environment with conditions of hypoxia, nutrient starvation, low pH and abundance of free radicals that induces endoplasmic reticulum (ER) stress and the unfolded protein response (UPR) in its cellular constituents. While the function of the UPR is to restore protein synthesis homeostasis, prolonged ER stress can activate MDSCs and promote reprogramming towards a more immunosuppressive and tolerogenic phenotype [116]. Influential work published by Condamine and colleagues found that intratumoral ER stress results in increased apoptosis of MDSCs in peripheral blood through TNF-related apoptosis-induced ligand receptor 2 (TRAIL-R) and caspase-8, thus facilitating expansion from the bone marrow [117]. This was surprising, given that MDSCs were previously thought to accumulate intratumorally due to evasion of apoptosis; instead, ER stress endemic to the TME causes a shortened life span and robust proliferation in the BM, leading to accumulation. As mentioned previously, expression of LOX-1 distinguishes PMN-MDSCs from neutrophils. Notably, addition of exogenous ER stress-inducers thapsigargin or dithiothreitol effectively converts neutrophils into LOX-1+ PMN-MDSCs [92]. Thus, the inherent ER stress-promoting environment of the TME has the capacity to both promote expansion from the bone marrow and spontaneously convert immune-activating cells into cells with an immunosuppressive phenotype.

Another mechanism by which MDSCs are recruited to the TME is through the uptake of tumor-derived exosomes (TEXs). TEXs are double-membraned extracellular vesicles containing various types of tumor antigens, proteins, lipids or nucleic acids that may be used for signal transmission between tumor and recipient cells. Beyond representing a potential non-invasive biomarker of both PCa diagnosis and prognosis [118], TEXs also promote the expansion and activation of MDSCs. The earliest report of this was from Xiang et al., who demonstrated that intake of TEXs by myeloid cells resulted in their differentiation into phenotypical and functional MDSCs with increased expression of COX2, IL-6, VEGF and ARG1 [119]. By neutralizing PGE2 and TGFβ with antibodies, they further showed that TEXs become enriched with PGE2 and TGFβ as the tumor progresses and that these molecules are responsible for the induction of MDSC. Hsp72 is expressed on the surface of TEXs and activates the STAT3 pathways in MDSC in a TLR2/MyD88-dependent manner via autocrine IL-6 production [120]. Interestingly, radiation therapy has been demonstrated to increase serum levels of Hsp72-containing exosomes in PCa patients [121]. Various TEX-derived miRNAs have been identified as responsible for MDSC expansion and activation in glioma [122], gastric cancer [123], chronic lymphocytic leukemia [124] and pancreatic ductal adenocarcinoma [125] cancers. However, each of these studies identified different miRNAs in different cancer types, and it is likely that PCa-derived TEXs will differ from those already described; further research in this regard is warranted to determine functional TEX-derived miRNAs and the mechanism of MDSC expansion/activation in PCa.

### 2.4. MDSCs in PCa Tumor Progression

The phenomena of intratumoral MDSC accumulation is an immunological hallmark in a variety of pathological conditions including solid cancers, and contributes to a potently immunosuppressive microenvironment, acquired resistance to cancer therapies, and poor prognosis. Indeed, multiple systematic review and meta-analyses have assessed the prognostic value of MDSC infiltration and have suggested its value as a clinical biomarker in various cancers [126,127,128]. While it should be noted that no such review has been produced for prostate cancer, strong correlations between MDSCs and cancer stages have been made [100,102], with one prospective correlative study demonstrating the ability of serum MDSC levels to distinguish between metastatic, localized and control patients [129].

An intimate crosstalk exists between MDSCs and tumor cells in PCa that drives castration resistance and promotes tumor progression. One such example of this is IDO, which is overexpressed not only by MDSCs but cancer cells themselves. Tumor-derived IDO has been shown capable of recruiting and activating MDSC in a Treg-dependent manner [130]. While MDSCs drive castration resistance through IL-23 [131], the development of castration resistance correspondingly mediates IL-8 secretion within tumor stroma that drives subsequent PMN-MDSC infiltration [115]. The generation of de novo therapeutic resistance also appears to invite MDSC infiltration; although whether this is a chicken or egg situation is questionable. For example, abiraterone and enzalutamide-resistant mCRPC patients have significantly higher levels of FGF, GM-CSF, IL-10 and IL-6, all known to recruit and activate MDSCs [93]. Chemotherapy treatment in lung cancer has been shown to promote exosomal release of miRNAs by MDSCs, which function to prevent their death and promote angiogenesis [93,94,95,96,97,98,99,100,101,102,103,104,105,106,107,108,109,110,111,112,113,114,115,116,117,118,119,120,121,122,123,124,125,126,127,128,129,130,131,132].

Another possible contribution that MDSCs may make to tumorigenesis is the production of neutrophil elastase, a proteolytic enzyme essential for neutrophil extracellular trap (NET) formation. Neutrophil elastase production is upregulated in a variety of cancers and may be responsible for several stages of tumor progression, largely through extracellular transactivation of EGFR and MAPK, resulting in ERK activation and regulation of ERK-dependent genes responsible for proliferation [133] Additionally, cleavage of tumor suppressors EMILIN1 [134] and p200 CUX1 [135] by neutrophil elastase results in their inactivation. Despite its name, neutrophil elastase is also produced by macrophages, lymphocytes and MDSCs. In PCa xenografts and prostate tumors of Pten-null mice, enzymatically active neutrophil elastase is produced by PMN-MDSCs that promotes tumor growth [136]. Thus, the increased levels of MDSC in prostate cancer disease progression is likely followed by increased neutrophil elastase expression, another tumor-enabling mechanism by MDSCs.

### 2.5. Mechanisms of Immunosuppression

PCa is characterized by T cell exclusion from the stroma, but accumulation and infiltration in peripheral margins [137], indicating a distinctly immunosuppressive TME. Thus, one hypothesis for the lack of response to immunotherapy in PCa is that T cells do not interact with cancer cells at all, making ICB a gratuitous feat. Those that do successfully infiltrate despite inhibitory migration signals face nutrient starvation, TCR nitration and direct inhibition through activation of immune checkpoints. The main immunosuppressive mechanisms include, but are not limited to, production of ARG1, iNOS, TGFβ, IL-10, COX2, sequestration of cysteine and depletion of tryptophan via IDO and a decrease in L-selectin by T cells. PMN-MDSCs are thought to be the prevalent subset in advanced stages of the disease and more readily infiltrate into the tumor stroma compared to the epithelium [105], enabling close cell-to-cell contact and paracrine signaling to be facilitated with T cells. One of the main immunosuppressive mechanisms utilized by MDSCs is the excessive production and secretion of ARG1, which is an enzyme that converts L-arginine into L-ornithine and urea [110]. Excessive production of ARG1 therefore leads to a rapid depletion of L-arginine available for local T cells, resulting in translational blockade and cell cycle arrest in G0—G1. L-arginine starvation is further exacerbated through expression of inducible nitric oxide synthase (iNOS) by MDSCs, which converts L-arginine into nitric oxide (NO), a compound which itself has immunosuppressive functions [109]. COX2 is an enzyme that is upregulated in MDSCs that produces prostaglandin E2 (PGE2), which acts in an autocrine manner to facilitate ARG1 production. Similarly, increased IDO expression depletes local levels of L-tryptophan and produces the immunosuppressive metabolite N-formulkynurenine, resulting in T cell tryptophan starvation, cell cycle arrest, immunological anergy and differentiation into Treg cells [91]. IDO expression is mediated by STAT3 yet not all MDSC express IDO, indicating that IDO expression possibly plays a supplementary role in T cell immunosuppression.

The generation of reactive nitrogen species (RNS) and ROS by MDSCs is a potent modulator of T-cell activity through the modification of the T-cell receptor (TCR)/peptide-bound major histocompatibility complex-I (pMHC-I) and immune-activating chemokines. Upregulation of NOX2 in MDSCs results in production of excessive superoxide, a reactive compound that rapidly reacts with H2O2, hydroxyl radical or hypochlorous acid leading to oxidative stress, apoptosis and inflammation [91]. Additionally, PMN-MDSCs and M-MDSCs overexpress eNOS and iNOS, respectively, both of which pr\oduce NO which reacts with superoxide to form peroxynitrite (ONOO−). MDSC-derived peroxynitrite nitrates tyrosine residues on the TCR, which increases conformational rigidity and leads to reduced binding capacity with pMHC-I of antitumor APCs [138]. Mechanistically, nitration causes the TCR and its co-receptor CD3ζ to dissociate, resulting in disruption of the complex, loss of responsiveness to pMHC-I, and inhibited activation of both CD8+ and CD4+ T cells [139]. Peroxynitrite can additionally nitrate the pMHC complex [140], the lymphocyte-specific protein tyrosine kinase (LCK) [141] and the pro-inflammatory chemokine CCL2 [142]. The known site of LCK nitration is Tyr394, and this modification results in a hindered ability to phosphorylate the CD3ζ complex and transmit T cell activation signals. Nitration of CCL2 reduces its ability to traffic T cells to the site of the tumor yet retains the ability to recruit myeloid cells.

MDSCs are also indirectly immunosuppressive through the modulation of other cells within the TME. MDSCs directly regulate both the development and clonal expansion of Treg cells, which is achieved through cell–cell contact and production of cytokines and enzymes such as IFNg, IL-10 and TGFβ and ARG1 [88]. They additionally promote TAM reprogramming to the immunosuppressive M2 phenotype through cell-to-cell contact that results in a switch from IL-12 secretion to IL-10 [143]. NK-derived IL-10 induces a positive feedback loop between MDSCs which respond by also secreting IL-10, and the lack of NK-activating IL-12 results in suppression of these cells. MDSCs maintain crosstalk with NK cells and inhibit their activities through the expression of TGFβ and interaction with NK-activating receptors, which significantly impairs degranulation capabilities and IFNg release [144].

Finally, MDSCs facilitate immune system anergy through the expression of PD-L1, which binds to PD-1 receptors on T cells, triggering cell cycle arrest and apoptosis. In colon cancer patients, 60% of circulating MDSCs are PD-L1+, and this number is significantly increased in the TME in vivo of tumor-bearing mice [145]. PD-L1 expression on MDSCs is exacerbated under hypoxic conditions, which causes HIF-1α to bind to a transcriptionally active hypoxia-regulated element in the PD-L1 promoter [146]. This hypoxia-mediated PD-L1 expression also renders MDSCs more immunosuppressive through increased expression of IL-6 and IL-10, although the mechanism behind this is not understood. Thus, hypoxic conditions within the TME not only facilitate MDSC activation but also immunosuppressive function by regulating expression of PD-L1 and facilitating immune checkpoints.

## 3. Targeting MDSC In Immunotherapy

There are a variety of methods that can be used to target MDSCs these are loosely grouped into four categories: (1) targeting MDSC immune regulatory properties, (2) targeting MDSC infiltration/activation (3) targeting MDSC development/maturation and (4) inducing MDSC apoptosis. A brief summary of the most pertinent pathways and compounds to targeting these pathways are herein discussed and summarized in Table 1.

### 3.1. Targeting MDSC Immune Regulatory Properties

The JAK/STAT3 pathway is a major regulator of many of the immunosuppressive mechanisms employed by MDSCs. It is well-described that MDSCs isolated from both mouse and human tumors display elevated levels of STAT3, and inhibition of this pathway results in significantly antitumor activity [104,147,148]. STAT3 regulates the expression of IDO, ARG1, IL-6, IL-10, IL-1β, VEGF and S100A8/A9 [91] among others, making this signaling pathway an attractive target for MDSC inhibition. MDSCs both synthesize and respond to S100A8/A9 in an autocrine feedback loop that signals through the NF-κB pathway that promotes intratumoral migration and accumulation [149]. The S100A8/A9 heterodimer is also involved in the formation of the NOX2 complex [150], thus potentiating the production of immunosuppressive ROS. STAT3 interacts with C/EBPβ to promote MDSC differentiation; in both C/EBPβ and STAT3-deficient cells, myeloid progenitors lose the ability to differentiate into functional MDSCs, even in the presence of G-CSF [151]. STAT3 additionally permits prolonged binding of C/EBPβ to the promoter of cell-survival protein Myc and is responsible for activation of other cell cycle-regulating proteins such as Bcl-Xl, survivin, Mcl-1 and cyclin D1. The STAT3 signaling pathway, which mediates cellular functioning in both subsets of MDSCs and mediates immunosuppressive functioning in the highly potent PMN-MDSC subset, is a tantalizing therapeutic target. Galiellalactone, a fungal-derived direct pSTAT3 inhibitor, was recently investigated for its ability to prevent PCa-induced generation by coculturing primary human monocytes in several ex vivo human prostate cancer lines [101]. The STAT3 inhibitor effectively prevented MDSC generation and inhibited IL-8 and GM-CSF secretion. Additionally, it could serve a duality of purpose in PCa, as it has also been previously shown to induce apoptosis in stem cell-like PCa cells [152], a subset of slow-growing cells that has been proposed to be responsible for tumor-regeneration and drug resistance. However, it is possible that galiellalactone is too broad-spectrum for use in PCa, a question that remains to be addressed in vivo. STAT3 is, importantly, not activated in non-malignant cells but is relied upon in the development of memory CD4+ and CD8+ T cells [153,154], emphasizing the need for STAT3 inhibition techniques that are selective towards MDSCs. One such strategy has been cleverly devised by Kortylewski et al. through the uptake of anti-STAT3 siRNA conjugated to an agonist of the endosomal toll-like receptor 9 (TLR9), which is expressed by cells of myeloid lineage [155]. The Kortylewski group then assessed the efficacy of this siRNA conjugate on ex vivo myeloid cell populations from healthy, localized and mCRPC PCa patients, and demonstrated that the conjugate is rapidly internalized by PMN-MDSCs and abrogates immunosuppressive activity and ARG1 expression [104]. Although TLR9 expression does occur in other cells of myeloid lineage such as plasmacytoid DCs and B cells, in cancer patients PMN-MDSCs represent the overwhelming majority of STAT3-regulated cells and increase with disease progression. Recently, the same group modified this method by conjugating the TLR9 agonist to chemically modified STAT3 antisense oligonucleotides (ASO), which improved the potency and nuclease resistance of the molecule [147]. Interestingly, CpG-STAT3ASO was taken up by both PCa tumor cells and MDSCs, resulting in the eradication of bone-localized prostate tumors in mice and alleviation of tolerogenic activity of PMN-MDSCs from human prostate cancers. With such a favorable potential safety profile and ability to eradicate tumors regardless of genetic background, the CpG-STAT3ASO represents a promising therapeutic avenue to MDSC-targeted strategies. Pre-clinical studies are still ongoing, and it remains to be determined how STAT3 silencing mechanistically inhibits MDSC function, i.e., whether it modulates immunosuppressive ARG1 expression or if it induces maturation into mature granulocytes, macrophages and dendritic cells.

The STAT1 pathway is responsible for IFNγ release, which inhibits PMN-MDSC maturation and enhances NO-mediated T cell suppression [111]. Both MDSC subtypes rely on STAT1 for immunosuppressive functioning, but only M-MDSCs rely on it for NO-mediated immunosuppression, and knockout of this pathway effectively results in downregulation of iNOS, ARG1 and T cell suppression in mice. Certain small-molecule inhibitors have been used to target STAT pathways with efficacy against MDSCs. However, in vivo, these compounds often lack efficacy and confer toxicity.

One of the most profound mechanisms of MDSC-mediated immunosuppression is the production of ROS and RNS, which not only enhances MDSC activity but induces antigen-specific immune tolerance of prostate tumor antigens. As such, pharmacological inhibition of iNOS and eNOS to prevent NO production as well as ROS scavengers could have a powerful antitumor effect. Certain drugs belonging to this category are NOS-inhibiting compounds such as phosphodiesterase-5 (PDE-5) inhibitors sildenafil and tadalafil, nitro-asprin, AT38 and antioxidant-producing compounds such as bardoxolone methyl, which induces NRF2-dependent antioxidant genes [156]. Lastly, ARG1 production may be attenuated through the inhibition of COX2 and COX2-derived PGE2; one such agent that achieves this is the highly selective and reversible COX2 inhibitor celecoxib.

### 3.2. Targeting MDSC Infiltration/Activation

Due to the overlap of factors involved in both accumulation and immunosuppressive function of MDSCs, many of the aforementioned techniques are likely to also inhibit MDSC expansion within the TME, such as STAT3 and COX2 inhibition. Other strategies include neutralizing MDSC-trafficking and promoting molecules through monoclonal antibodies or small-molecule inhibitors against VEGF, IL-1B, semaphorin 4D and CXCR2 [111]. Anti-VEGF treatment is already used to reduce neovascularization in various cancers; however, such efficacy may also be attributable to MDSC inhibition. Various lines of evidence indicate that VEGF neutralization with the mAb bevacizumab reduces PMN-MDSCs in the circulation of multiple cancer types and mouse models. However, recent evidence suggests that this compound enhances MDSC intratumoral recruitment by inducing hypoxia and GM-CSF expression [157]. Monoclonal antibodies against CXCR2 have been demonstrated to inhibit MDSC infiltration and improve ICB efficacy [158]. One recently proposed CXCR1/CXCR2 inhibitor is orally bioavailable SX-682, which apparently selectively inhibits intratumoral PMN-MDSC infiltration without altering CXCR1+ macrophages [159]. CXCR2 interacts with both of the PCa-secreted cytokines IL-8 and CXCL6. By use of the CXCL2 antagonist SB255002, Wang et al. demonstrated significant reduction in tumor burden and prolonged survival of mouse models in vivo [103]. Furthermore, they demonstrated the potent antitumor ability of the CXCR5/CRCX2 pathway that is regulated by Hippo-YAP1 signaling pathway, which could represent a potential pharmacological target.

### 3.3. Targeting MDSC Development/Maturation

The immunosuppressive capability of MDSC is associated with their undifferentiated state and inducing terminal differentiation into macrophages, DCs and granulocytes is one method of alleviating their suppressive functions. Compounds that promote MDSC maturation include bisphosphates, vitamin D3, vitamin A, all-trans retinoic acid (ATRA), CpG oligonucleotides and IL-12 [111]. Bisphosphates work by inhibiting isoprenylation post-translational modifications in the bone marrow, which leads to osteoclast apoptosis [156]. Given that MDSC expansion is the result of intratumoral TRAIL-R/caspase 8-mediated apoptosis and robust proliferation in the bone marrow [117], such a compound should effectively prevent MDSC accumulation in both tumor and blood and pre-empt immunosuppressive functioning. Another notable compound for the inhibition of MDSC maturation is ATRA, which has shown efficacy in both mouse and humans through ROS depletion, which MDSCs rely on to remain undifferentiated [96,97]. ATRA functions by upregulating glutathione synthase, resulting in increased levels of the antioxidant glutathione, which scavenges ROS and potentiates MDSC differentiation [160].

### 3.4. Inducing MDSC Depletion

Depletion of MDSCs by means of inducing cytotoxic death or by MDSC-specific antibodies to selectively eliminate MDSCs while preserving healthy cells remains challenging. Some drugs in this category include chemotherapy agents such as cisplatin, paclitaxel, doxorubicin, trabectedin, gemcitabine, docetaxel and 5-fluorouracil (5-FU), receptor tyrosine kinase (RTK) inhibitors such as nilotinib, dasatinib, sorafenib, ibrutinib, and sunitinib [111], which would serve a dual purpose of targeting both cancer cells and MDSCs. Although each of these compounds have demonstrated the ability to deplete MDSCs in vivo, with some entering clinical trials for certain cancers, cytotoxicity remains an important obstacle. The best use of cytotoxic compounds is probably at low dosages in an adjuvant combinatorial setting alongside immunotherapy and/or ADT [161,162].

Murine MDSCs can be exclusively characterized through their concurrent expression of CD11b and Ly6C (M-MDSC) or Ly6G (PMN-MDSC) [110]. As such, mouse prostate cancer models can be easily depleted of circulating MDSCs with the well-characterized monoclonal antibody Gr-1, which recognizes both Ly6C and Ly6G epitopes. In mouse models, Gr-1-mediated MDSC depletion successfully resulted in CD8+ T cell expansion and significant tumor weight reduction [163]; warranting further research into how to deplete human MDSCs in a similar manner. To this extent, the monoclonal antibody DS-8273a agonist of death receptor TRAIL-R2 was demonstrated to be selected towards MDSCs [148] and has launched clinical trials in combination with anti PD-1 in melanoma and colorectal cancer. Unfortunately, human MDSCs do not possess such a unique surface marker rendering their depletion using a single targeting antibody difficult. Qin et al. however, managed to develop a novel peptibody composed of a newly identified MDSC-binding peptides (H6) fused to the Fc portion of mouse IgG called Pep-H6 [164]. Pep-H6 targets S100A9 that is displayed on the surface on MDSCs and is secreted in an autocrine fashion to maintain their accumulation in tumors and was demonstrated to successfully deplete both MDSC subtypes in vivo in blood, spleen and tumors, resulting in inhibition of tumor growth and survival benefit [103]. Another inhibitor of S100A9 is tasquinimod, which is a small-molecule oral agent instead of an antibody conjugate. Tasquinimod was previously under phase III clinical investigation for use in mCRPC as a single agent but did not significantly prolong survival [161]. Despite this result, there was still promise for this drug as a combinatorial agent in immunotherapy, and a phase II trial was launched in combination with sipuleucel T. However, due to a lack of funding, the study remains unfinished. Given that abrogation of S100A9 is sufficient to deplete MDSCs in vivo with Pep-H6, it is reasonable to hypothesize that similar effects would be attained with tasquinimod treatment, and that immunotherapy efficacy would be improved with low-dose combinatorial use of this compound.

### 3.5. Combinatorial Strategies

MDSCs represent one essential cog in a larger machine and would be best targeted in combination with immunotherapy to simultaneously relieve potent immunosuppression while actively initiating an antigen-specific immune response. Currently, the most noteworthy example of this in the field of prostate cancer is a set of elegant experiments by Lu and colleagues, who generated a novel mCRPC mouse model with the genotype PB-Cre+ PtenL/L p53L/L Smad4L/L mTmGL/+ LSL-LUCL/+, resulting in a 4-fold increase in number of PCa-bearing mice that can be manipulated to become castration-resistant [162]. They then used this novel model to assess antitumor effects of ICB and the MDSC-targeting agents cabozantinib (a TKI) and BEZ235 (a PI3K/mTOR inhibitor) alone and in combination. Neither ICB nor MDSC-targeting agents were efficacious when used as a monotreatment but induced a robust synergistic response when combined. Both cabozantinib + ICB and BEZ235 + ICB resulted in inhibition of proliferation and induced apoptosis of the primary tumor, notable cytokine changes and significant reduction in lymph and lung metastases. However, this was not observed for another MDSC-targeting TKI, dasatinib. 

Production of RNS and peroxynitrite is a potent immunosuppressive mechanism utilized by both types of MDSC through different pathways. Therefore, one effective targeted strategy would be to neutralize peroxynitrite, thus preventing nitration of the TCR/pMHC complex and restoring T cell activation. Feng et. al. investigated this strategy in a combinatorial setting with ICB in the form of both anti-CTLA4 and anti-PD1 antibodies and determined that RNS neutralization with uric acid (UA) sensitized CRPC to immunotherapy [141]. Like Lu et al., a synergistic response was achieved when a combinatorial strategy was used that was not observed using either ICB or UA alone, with a six-fold increased CD8+/Treg ratio compared to controls. By depleting MDSC with anti-Gr-1 antibody, they further demonstrated that MDSCs are responsible for a large majority of 3-NT on both PCa and CD3+ T cells, rendering this an effective MDSC-targeted strategy.

Based on the finding that IL-8 drives PMN-MDSC intratumoral infiltration, Lopez-Bujanda et al. tested the triple combination of ADT, anti-CTLA-4 and anti-CXCR2 (the receptor to IL-8) on the MyC-CaP mouse model [115]. Compared to ADT combined with anti-CTLA-4 alone, this triple combination resulted in delayed castration resistance, reduction in tumor volume and significantly increased survival. This response was not attributed to any increase in T cell infiltration or decrease in Treg infiltration but instead to an increase in polyfunctional CD8+ T cells in lymph and spleen; indicating that MDSCs had been effectively targeted through this strategy. These findings have launched a phase Ib/2 trial that is currently underway to investigate the efficacy of ADT, anti-IL-8 and anti-PD-1 in men with mCRPC. Notably, the Lopez-Bujanda study found IL-8 to be upregulated post-ADT, suggesting that AR re-activation mediates IL-8 secretion by PCa cells that drives PMN-MDSC infiltration. Thus, a potentially better point of combinatorial intervention may be during ADT, as opposed to the onset of mCRPC.

MDSCs are highly stimulated under chronic hypoxic conditions through ER stress and activation of the UPR response. Evofosfamide is a hypoxia-activated, hypoxia-disrupting prodrug which was recently tested in combination with anti-CTLA-4 and anti-PD-1 in TRAMP mice with profound antitumor results [165]. MDSC frequency and recruitment were significantly inhibited, allowing T cells to infiltrate and proliferate at a 2-fold increased rate in the formerly hypoxic areas. As such, mice exhibited long-term antitumor effects, with little to no observable tumor burden up to 3 months after therapy. This study has launched a phase I clinical trial of evofosfamide in combination with anti-CTLA-4, although the status of this trial is unknown at the time of writing.

Of special interest is the QuEST1 phase I/II clinical trial that is currently underway, which aims to provide an expedited investigation of a four-pronged combinatorial strategy composed of an antitumor vaccine, immune checkpoint blockade, NK cell activating-agent, and MDSC-targeted inhibitor [166]. In this case the antitumor vaccine targets the tumor-specific antigen brachyury, which is an embryonic transcription factor that becomes re-activated in malignant cells to promote tumor migration through the epithelial–mesenchymal transition (EMT) [167]. The agent used for ICB is the innovative fusion protein TGFβ TRAP/anti-PD-L1 antibody (M7824) which serves the dual purpose of sequestering TGFβ and blocking PD-L1 interactions. ALT-803 is an IL-15 and IL-2 superagonist which effectively improves NK cell and T cell function and frequency, and the IDO inhibitor epacadostat should neutralize both PCa- and MDSC-derived IDO. The trial aims to test the brachyury-based vaccine in three arms: (i) vaccine + TGFβ TRAP/anti-PD-L1, (ii) vaccine + M7824 + ALT-803, and (iii) vaccine + M7824 + ALT-803 + epacadostat. It should be highly informative to see the difference between these various arms, particularly between arm (ii) and (iii), which incorporates the use of an MDSC-targeted strategy.

## 4. Other Important Cells and Regulator Contributing to the Overall Immunosuppression or Lack of Antitumor Immune Response

### 4.1. ADRB2

Androgen resistance is associated with the recruitment of myeloid-derived suppressor cells (MDSCs) into the tumor microenvironment (TME) and the consequential establishment of an immunosuppressive TME. MDSC recruitment is driven by local inflammation and stress/depression which induces neuropeptide Y secretion from prostate cancer cells [168]. In addition, prostate adenocarcinoma extensively treated with ADT or radiation therapy [169] transdifferentiates into an aggressive form of CRPC termed neuroendocrine prostate cancer (NEPC) without undergoing pluripotent cell transition [170]; a phenomenon poorly understood. While some cases of NEPC can arise de novo, the vast majority are treatment-related (tNPEC) [170,171].

NEPC is a poorly defined clinical phenotype of aggressive disease and causes approximately 10–25% of prostate cancer-related deaths [171,172]. Neuroendocrine-like cells (NECs) are characterized by loss of AR expression [171,172], activation of the β-adrenergic receptor (ADRB2), elevated levels of stem cell and neuroendocrine markers as well as resistance to hormonal therapies. Sympathetic nerves are crucial in PCa generation and growth, and perineural invasion (PNI) is a condition where tumor spreads through the nerves tissue and correlates with poor prognosis. ADRB2 is the most abundant receptor for sympathetic signals in prostate luminal cells [169]. Interestingly, meta-analysis has revealed that psychological depression/chronic stress is prevalent in men with prostate cancer and patients with these conditions can deliver increased adrenergic signals via sympathetic nerve fibers, which then act via β-adrenergic receptors expressed on cancer cells, thereby promoting cell proliferation [172].

The prostate has an abundant nerve supply, and the contribution of sympathetic nerves is crucial for differentiation and secretion by luminal cells [170,173]. ADRB2 is the primary receptor through which the sympathetic regulation takes place [169,170]. ADRB2 is a G-protein coupled receptor (GPCR) which is stimulated when bound with stress-facilitating catecholamines [174]; with more affinity towards Adrenaline (Ad) compared to noradrenaline (NAd) via adenyl cyclase pathways with increased cyclic adenosine monophosphate (cAMP), protein kinase A (PKA) and cAMP response element-binding protein (CREB) [170,175]. In physiological conditions this functions to regulate the response-reaction and energy expenditure in the face of perceived danger [174]. In the PCa TME, these adrenergic receptors are found on luminal cells and are increased in cancer cells compared to benign conditions [170]. These are not only stimulated by Ad from the adrenal medulla and NAd from adrenergic nerves, but also from Ad/NAd release from macrophages, lymphocytes, and neutrophils [170,174,176]. Previous studies on several cancer types reported ADRB2-mediated apoptosis, metastasis, angiogenesis, and therapy resistance, and it has been hypothesized that targeting this receptor would benefit CRPC-related adversities [170,176]. Non-selective beta blockers, such as propranolol, can have detrimental effect on people with low blood pressure and therefore a more beta2-targeted blocker such as ICI-118551 would be safer. Moreover, the number of ADRB2 is different between patients, within the tumor itself as well as during different disease stages which could explain the heterogenous effect of these drugs. In a pre-clinical study design consisting of tissue and mouse models, ADRB2 expression in hormone naïve PCa was found to be elevated but diminished with increasing tumor grade [169,170,171,172]. In an ADT-treated environment, NECs appear to promote androgen insensitivity and severity of the tumor resulting in poor prognosis [177]. Despite the minor contribution from pre-existing nerve-like cells, NECs involved in malignancy are believed to mainly emerge from neuroendocrine transdifferentiation (NED) [170,174,177].

Plausible causes behind NED include the prolonged application of ADT, radiation therapy, and it has been evident that ADRB2-mediated catecholamines induce NEC proliferation in AR-dependent and -independent manner [170,177]. In treatment naïve cell lines expressing high level of ADRB2, NED did not occur, only the neuronal type cells were increased [170]. Moreover, tumor cells with high levels of ADRB2 before starting ADT treatment were found to transdifferentiate into NED more frequently after ADT treatment compared to those with the low level of the proteins [171]. If this were to be confirmed in human PCa cells, then it would become necessary to assess the level of ADRB2 expression prior to ADT treatment and taken into consideration for additional treatment such as ADRB2 inhibitors (ADRB2i).

Ongoing clinical trials on beta-adrenergic blockers include the oral administration of Etodolac and Propranolol (NCT01857817-phase II), beta-blockade using Carvedilol for prostate adenocarcinoma patients before prostatectomy (NCT02944201-phase II), and Propranolol Hydrochloride treatment in patients undergoing surgery (NCT03152786-phase II) [169,178]. Unravelling the pathways and molecules involved are essential to incorporate in this arm of studies as well as considering efficacy, safety and the patient cohort that would benefit from the ADRB2 blockers.

### 4.2. Other Important Regulators of PCa Progression—Histone Deacetylase (HDAC)

The development of CRPC and androgen insensitivity are significant shifts in the progression of PCa, the causes of which are currently being investigated. In this context, mediators of epigenetic changes related to PCa are presently being examined, with HDACs among the prime areas of inquiry. Chromatin, made up of DNA, is wrapped around histone proteins, and chromatin remodeling is regulated by acetylation, phosphorylation, and methylation of the histone [179]. Histone acetyltransferases (HATs) attach acetyl groups to DNA and loosen the histone wrap allowing the transcription factors to communicate the DNA and regulate the gene expressions. In contrast, HDAC removes the acetyl group from the histone, leaving DNA compressed onto the histone and hinders the attachment of transcription factors to DNA [180]. These alterations lead to gene silencing and the suppression of the associated expression. In physiological conditions, HDACs function as regulators of chromatin structure and functions [179,180]. There are four main groups of HDACs known at present with a total count of 18 isoforms. Classes I, II and IV are called as “Classical HDACs” and have a curved tubular pocket where the zinc ion is attached and mediate the activities [179,180,181].

Further, HDACs are involved in non-histone protein functional regulation in addition to the chromatin modulation, and in cancerous conditions, HDAC-mediated epigenetic regulation promotes neoplasm [179,181]. Activating HAT or deactivating HDAC may resolve the abnormality in some conditions such as the gene expression of AR in PCa [182,183]. From a technological point of view, however, stimulating an enzyme is more challenging than suppressing it, and HDAC inhibition has therefore, been preferred in therapeutic interventions over HAT induction [180]. HDAC inhibitors (HDACis) can be classified as PAN inhibitor or selective inhibitor depending on their specificity as to several classes of HDACi or a specific class [179].

Many HDACis have already been FDA approved for the treatment of various cancers Vorinostat (SAHA) for the treatment of cutaneous T-cell lymphoma (CTCL) [184], Romidepsin for the treatment of CTCL and T-cell lymphoma (PTCL) [185], Panobinostat, for the treatment of multiple myeloma [186], Belinostat for the treatment of relapsed or refractory PTCL [187], while Valproic acid and Entinostat (MS-275) are currently in various clinical trials being used either alone or in combination with other form of cancer therapies [188]. Some of these are now being investigated for the treatment of prostate cancer. Histological analyses tracing the progression of PIN have shown that class I, II and IV HDACs are upregulated in PCa, suggesting their early occupation in the tumor. Specifically, HDAC1 and HDAC4 were found in increased numbers in CRPC [189,190]. Further, scientists have shown that expressions of HDAC2 is inversely correlated to PSA relapse-free survival, specifically in high Gleason Grade patients [189]. Elevated counts of HDAC in PCa was found to be further increased in metastatic cancer compared to non-metastatic PCa [191] and HDACs are key mediators of the AR axis in recurring PCa. Inhibiting HDACs is not only a possible way to induce tumor cell death but the treatment also appears to be less toxic towards normal cells [192]. There are currently several phases I and II trials ongoing using HDACis for PCa as monotherapy or as combination therapy (Table 2).

Targeting androgen synthesis/pathways (Figure 2) has been the prime treatment for PCa as cancer cells rely vastly on androgen for survival and proliferation. However, the majority of recurrent prostate cancers are neither completely hormone refractory nor androgen independent but are rather dependent on the AR signaling axis [209] and alternate AR pathways have been found to support PCa growth through the reactivation and targeting of AR [190,210]. Previous studies have shown some of the HDACis such as SAHA and dacinostat efficiently suppressed AR regulated transcriptional activities in PCa tissues, and more so effectively in AR-positive cancers [200,211]. These HDACis influence the AR axis through inhibition of heat shock protein-90 (HSP-90), AR transcription and transcription of AR target genes [211]. Further HDAC1 and HDAC4 were found to contribute to PCa proliferation and CRPC, respectively [190]. Other than direct targeting AR axis, there are several other molecules and pathways HDACis target and produce a decline in growth, proliferation, and trigger apoptosis. Recently an in vitro study displayed the ability of HDACis, SAHA and entinostat to improve the immunogenicity of PCa cells that may alter the immune-evasion phenotype of PCa. The experiment provides a rationale to incorporate HDACis with immunotherapy [212].

## 5. Concluding Remarks

The advent of immunotherapy has been one of the greatest achievements to science and anticancer therapies in the recent decades and continues to hold great potential for prostate cancer. It is abundantly clear that PCa possesses a distinctly immunosuppressive profile that, unlike other solid cancers, somehow evades immunotherapy to a more effective extent. This only highlights the need to better understand and target the key immunosuppressive cells to alleviate this potent immunosuppression within the TME and increase immunotherapy efficacy. Recent studies involving combinatorial MDSC-targeted therapy have not only substantiated the hypothesis that MDSCs represent one of the major obstacles in PCa immunotherapy but have also demonstrated that MDSC depletion restores intended immunotherapy outcomes.

One potential contributing factor to the perceived lack of efficacy of immunotherapy is the fact that it is only ever attempted in late-stage disease after alternate treatment options have been exhausted. Because disease progression is most often indolent, current treatment strategies aim to preserve the quality of life over aggressive treatment. However, by the time mCRPC has developed, multiple signaling pathways have been de-regulated and significant treatment resistance has been generated. Docetaxel is the primary line of treatment for mCRPC, and the average age of these patients is 70 years of age [47]. Thus, patients often receive immunotherapy in a pre-immunocompromised state, due to both age and chemotherapy pre-treatment. Additionally, circulating levels of M-MDSCs are normalized following surgical removal of the primary prostatic tumor [99], yet resurge once metastatic disease develops [100,101]. This direct link between elevated MDSC levels and presence of a primary tumor and/or metastases indicates that the optimal time point for MDSC-therapeutic intervention would be following prostatectomy, when levels have been normalized and are therefore minimally suppressive. This time point would also pre-empt the onset of castration resistance, which both drives PMN-MDSC infiltration through IL-8 [115] and is driven by MDSCs through IL-23 [131]. Therefore, the maximally beneficial setting for combinatorial immunotherapy would be as an adjuvant along with, or shortly after, ADT treatment.

Another important factor to consider is the two distinct subpopulations of MDSC, and their frequency and potency in various bodily locations. Despite many contradictory studies assessing the two subpopulations, relatively few have divulged a comprehensive understanding of the mechanistic role of each subtype in PCa. Given that the two subtypes rely on different signaling pathways to elicit different methods of immunosuppression, the question remains whether targeting only one pathway or subtype is enough to achieve therapeutic benefits. One such example is the STAT3 pathway, which both PMN- and M-MDSCs use but heavily governs immunosuppressive functioning in the PMN subset. In tumor-bearing mice, STAT3 inhibition resulted in MDSC depletion in spleens, but not tumors [87], leading to the notion that the signaling pathways governing these cells and their differential downstream effects are critical in their immunosuppressive capacity. Additionally, the abundance and potency vary by bodily location and disease stage. The conclusion that this review has reached is that in PCa, PMN-MDSCs appear to predominate in intratumoral and lymphoid populations and are possibly more potent than M-MDSCs, although this is debated. Such a lack of knowledge in this respect will be a major obstacle in determining the most effective MDSC-targeted therapeutic strategy.

**Figure 5:**
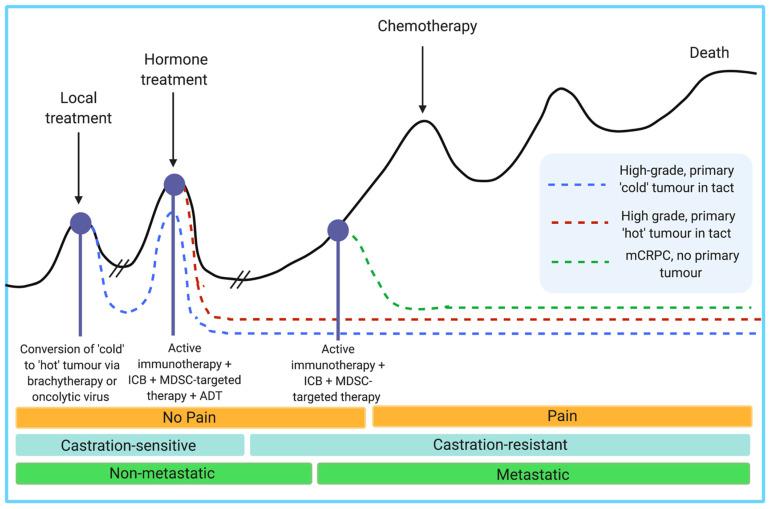
Proposed sequence of combinatorial immunotherapeutic intervention for different states of PCa. Black line represents the typical disease progression and arrows indicate currently used therapeutic interventions, which ultimately result in death for the majority of mCRPC patients. Dotted lines represent suggested points of intervention where combinatorial immunotherapeutic methods may be most impactful for different types of patients. Active immunotherapy refers to Sipuleucel-T or antitumor vaccine.

There are three types of high-risk patients for which the combinatorial strategy might differ: (i) those with a high-grade intact primary tumor (i.e., recently diagnosed and has not undergone RP) that is immunologically ‘cold’, (ii) those with a high-grade untreated disease that is immunologically ‘hot’, and (iii) those who have had the primary tumor removed and whose disease has progressed to mCRPC despite ADT treatment (Figure 5). For the first group wherein, there is access to the primary tumor, the first line of treatment should be broad immunological activation to a ‘hot’ tumor through brachytherapy [213] or oncolytic virus [214]. Once immune infiltration is permitted, an active immunotherapy treatment such as sipuleucel-T or an antitumor vaccine such as our mutated PAP-derived vaccine would be useful to generate antigen-specific immune responses against tumor antigen-expressing PCa cells. Finally, ICB (both anti-CTLA4 and anti-PD1 antibodies) and MDSC-targeted therapy could then be utilized to break immunological tolerance and permit CD8+ cytolytic activity on PCa cells. For the groups (ii) and (iii), immunological activation is either not necessary or not accessible via a primary tumor, so the starting point for these patients would be active immunotherapy, ICB and MDSC-targeted therapy. Groups (i) and (ii) additionally benefit from receiving ADT along with immunotherapeutic approaches as they are castration sensitive.

Beyond targeting the cells of the tumor and the immune system, a comprehensive strategy that takes patient psychology and microbiome into account could be necessary to generate robust and long-lasting clinical responses. Following the development of castration resistance, 25% of tumors will transform into an aggressive form of CRPC called NEPC wherein adenocarcinoma cells transdifferentiate into neuroendocrine-like cells and express the β2-adrenergic receptor (ADRB2) [215]. Depression, which is a neuroendocrine disorder, may therefore either be the cause or the result of neuroendocrine transdifferentiation, resulting in poor prognosis. Indeed, depression is significantly associated with mortality risk in mCRPC patients [216], and chronic stress promotes tumorigenesis through the activation of the ADRB2 by adrenaline, which mediates antiapoptotic pathways in PCa cells [217]. Depression has been recently demonstrated to induce neuropeptide Y expression by PCa cells through sympathetic activation, resulting in IL-6 release from TAMs and subsequent intratumoral MDSC infiltration [168]. Curiously, a meta-analysis of 16,825 PCa patients has previously associated the use of beta-blockers with reduced prostate cancer-specific mortality [172]. The available evidence indicates there is a potential benefit of pharmacologic intervention of adrenergic pathways and/or professional counselling, lifestyle habits, etc., for PCa patients at risk of depression, which may aid in ablation of MDSC-derived immunosuppression.

In line with this holistic therapeutic approach is consideration of the host microbiome and its effects on tumor progression. The relationship between the host gastrointestinal microbiome (which contains 10^13^–10^14^ microorganisms [218]) and antitumor immunity is particularly relevant in PCa patients who are often >50 years of age, and therefore likely present with lower bacterial load and diversity. Given that PCa progression is heavily influenced by chronic inflammation, it is no surprise that dynamic changes to a more pro-inflammatory microbiome, enriched in *Bacteroides* and *Streptococcus* spp. has been observed in PCa patients [219]. GI bacterial infection alone is sufficient to enhance prostate intraepithelial neoplasia (PIN) and microinvasive carcinoma [220]. This adjacent immune activation likely exacerbates the chronic inflammatory environment of the prostate tumor through ROS and RNS-mediated cell damage, which is known to directly contribute to the development of PIN and adenocarcinoma [221]. An added layer of complexity is the developing knowledge of the prostate tumor microbiome, which is no longer considered a sterile environment. Microbiome profiling of PCa tumor tissue has revealed to be enriched in Propionibacterium and *Corynebacterium* spp., which may be responsible for tumor progression by modulating host immune responses and ECM composition [222,223]. Curiously, eradication of the gut intestinal microbiota through antibiotic use or gnotobiotic organisms renders CTLA-4 treatment ineffective, a phenomenon that can be salvaged through supplementation with *B. fragilis* [224]. Little is known of beneficial species in the context of PCa. However, Lactobacillus rhanosus GG (LGG) is often used as an adjuvant for colorectal cancer treatment for its anti-inflammatory properties. Therefore, probiotics such as LGG, *B. fragilis* or other species may represent a useful adjuvant in therapeutic approaches to PCa.

**Figure 6:**
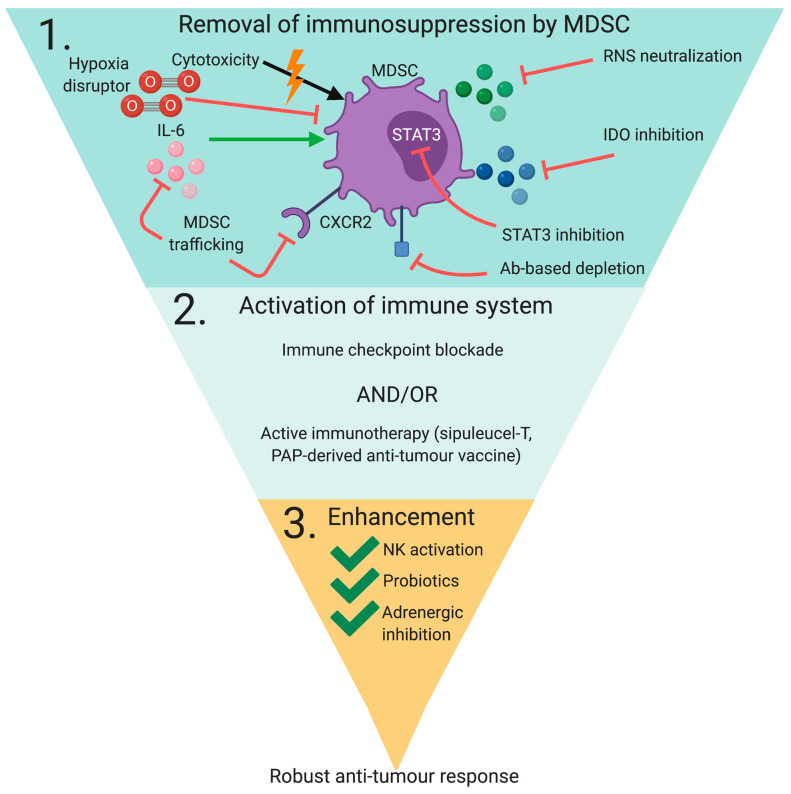
Comprehensive immunotherapeutic therapeutic strategy for the treatment of advanced prostate cancer.

To conclude, prostate cancer is a dynamic disease with a complicated and poorly understood etiology that likely requires a multipronged approach incorporating MDSC-targeting, immunotherapy, probiotics and inhibition of adrenergic pathways for clinical efficacy (Figure 6). Additionally, the combinatorial strategy should differ depending on the immunological status of the tumor and whether disease has progressed to mCRPC, which has an influence on the remaining available treatment options and prognosis. Further inquiries are warranted in determining the most effective MDSC-targeted treatment and indeed the most effective combinatorial strategy.

## Figures and Tables

**Table 1:** Synopsis of clinical trials involving singular and combinatorial MDSC-targeting agents for the treatment of prostate cancer.

Trial Identifier	Compound(s)	Mechanism of Action	Phase Reached	Cohort	Therapy Type	Outcome
**IL-6-Mediated Pathways**						
*N/A [Karkera]*	CNTO 328/Siltuximab	IL-6 chimeric monoclonal antibody	Phase I	Organ-confined PCa, *n* = 20	Monotherapy	Favorable preliminary safety profile with evidence of decreased activation of IL-6-mediated signalling pathways.
*NCT00401765*			Phase I	mCRPC, *n* = 40	Polytherapy (+docetaxel)	Promising PSA results but high toxicity.
*NCT00433446*			Phase II	mCRPC, prior taxane therapy, *n* = 53	Monotherapy	Poor efficacy, IL-6 increased dramatically post-treatment.
*NCT00385827*			Phase II	mCRPC, prior docetaxel therapy, *n* = 106	Polytherapy (+ mitoxantrone/prednisone)	Well tolerated, but no improvement in survival outcome.
**CXCL5/CXCR2-Mediated Pathways**						
*NCT03177187*	AZD5069	CXCR2 antagonist	Phase I/II	mCRPC	Polytherapy (+enzalutamide)	Currently recruiting.
***Targeting S100A9***						
*NCT01234311*	Tasquinimod	S100A9 inhibitor	Phase III	mCRPC, *n* = 1245	Monotherapy	No improvement in survival outcome.
**Combinatorial Immunotherapeutic Approaches**						
*NCT03098160*	Ipilipmumab/anti-CTLA4, Evofosamide	ICB, Hypoxia disrupting prodrug	Phase I	Prostate and other cancers	Combinatorial	Recruiting. Current recruitment status unknown.
*NCT03689699*	Nivolumab/anti-PD-1, BMS-986253/anti IL-8	ICB, fully monoclonal antibody against IL-8	Phase I/II	Hormone-sensitive PCa		Currently recruiting.
*NCT03493945*	BN-Brachyury, M7824, ALT-803, Epacadostat	Anti-tumour vaccine, TGFβ TRAP/PD-L1, IL-15 agonist, IDO inhibitor	Phase I/II	CRPC		Currently recruiting.
*NCT02159950*	Tasquinimod, Sipuleucel T	S100A9 inhibitor, active immunotherapy	Phase II	mCRPC		Not completed due to lack of funding.

**Table 2:** Studies that targeted HDACs in PCa and their details.

HDACi	Targeted HDAC	Test Model	Strategies and Results	Reference
Dacinostat (LAQ824)	Pan (Class I, II)	Androgen-sensitive and -independent cell line	Acetylation of HSP-90 and concurrent suppression in ATP binding leads to proteasomal degradation of AR by dissociation of AR and HSP-90. HDACi is believed to target either HDAC6 or HDAC10.	[193]
		Phase I trial	Reduced levels of HSP-90 protein as an indication of the inhibition by the HDACi.	[194]
Suberoylanilide hydroxamic acid (SAHA)/Vorinostat	Pan	cell lines and xenografts (CWR22 nude mice)	Acetylation of HDAC 3 and 4 resulted in tumour regression, apoptosis, and growth arrest in cancer cells.	[191]
		Phase II	No significant outcome, progression-free disease in 2 patients out of 27 patients (NCT00330161).	[195]
Belinostat (PXD101)	Pan	Combination treatment with docetaxel on xenograft mice	Inhibition of HDAC 6, stabilizing tubulin acetylation, regulating antiapoptotic proteins including BclXL to induce death of hormone-refractory cancer cells resulting reduced tumour volume.	[192]
SAHA derivative (MHY219)	HDAC 1, 2, 3 and 4	metastatic in vitro tissues	Increased apoptosis of PCa	[196]
			Increased tissue inhibitor of metalloproteinase-1 (TIMP1) and associated mRNA levels. Reduced levels of MMP1 and MMP2. The regulator of MMPs, HDAC1 activity was also inhibited and limited cell migration.	[190]
Panobinostat (LBH589)	Pan	In vitro and in vivo studies with Zoledronic acid (Zol)	Panobinostat relieves drug resistance to Zol and results in cell arrest and apoptosis of tumour cells.	[197]
		Phase I trial, oral administration to CRPC patients along with docetaxel	≥50% drop of PSA in 5 out of 8 patients.	[198]
		Phase II trial, IV administration to CRPC patients after receiving chemotherapy.	No significant outcome (NCT00667862).	[199]
Ivaltinostat(CG200745)	Pan	Combination treatment with docetaxel in vitro and in vivo models	The inhibition through entrapping ARs in microtubules [tubulin acetylation] and stabilize the microtubule. Results include downregulated full-length AR and AR splice variants, PSA, Bcl2 proteins and reduced cell viability.	[200]
Nicotinamide	Sirt1	In vitro and in vivo studies	Silencing of SIRT1 with nicotinamide and genetical suppression by sirtinol result in growth arrest and apoptosis of PCa cells	[183]
Sirtinol	Sirt1/2			
VPA	Pan Class I, IIa HDACs	Administrated with drinking water in xenograft mice model	Cell cycle arrest, apoptosis and reduced angiogenesis and reduced levels of proliferating cell nuclear antigen(PCNA).	[180]
		Metastatic PCa cell lines	Only altered metastasis tissue, increased metastasis suppressor gene N-myc downstream-regulated gene (NDRG1), reduced metastasis	[201]
Entinostat (MS275)	Class I HDACs	RM1-CRPC rodent modal (Resemble bone metastasis)	Induction of tumour intrinsic type 1 interferon (TI1IFN) correlated with T cell responses displaying the increased immunogenicity	[202]
		In vitro analysis and DU145 xenograft mice	Inhibition of HDAC 4, Attenuation of DNA damage repair, enhanced radiosensitivity and tumour delay.	[203,204]
		CRPC-MyCaP mice were administrated SurVaxM	Reduced FOXP3 expression and expansion of CD8+ T cells. Un changed Treg levels.	[182]
Romidepsin		Phase II trial	In a small portion, RECIST and ≥ 50% drop of PSA (NCT00106418).	[205]
Pracinostat (SB939)	Pan Class-I, -II and -IV	Oral administration, Phase II trial mCRPC patients	Low drug toxicity, no significant outcome (NCT01075308).	[206]
Tasquinimod	HDAC3/4	Phase II and III trials	Angiogenesis, MDSC suppression which shown progression-free disease but no improvement in survival of the patients (NCT01234311).	[207,208]

## Data Availability

All Figures were created by BioRender.com (accessed on 8 March 2021).

## Abbreviations

5-FU	5-fluorouracil
Ad	Adrenaline
ADRB2	β-adrenergic receptor
ADRB2i	ADRB2i inhibitor
ADT	Androgen deprivation therapy
APC	Antigen-presenting cell
AR	Androgen receptor
ARE	Antioxidant response element
ARG1	Arginase 1
ATRA	All transretinoic acid
CAB	Combined androgen blockade
CAF	Cancer-associated fibroblast
CREB	cAMP response element-binding protein
CRPC	Castration-resistant prostate cancer
CTL	Cytotoxic T-lymphocyte
DAMPs	Danger-associated molecular patterns
DC	Dendritic cell
DHEA	Dehydroepiandrosterone
DHT	Dihydrotestosterone
DRE	Digital rectal examination
ECM	Extracellular matrix
EMT	Epithelial–mesenchymal transition
EMC	Epithelial-mesenchymal cell
ER	Endoplasmic reticulum
GM-CSF	Granulocyte-macrophage colony-stimulating factor
G-CSF	Granulocyte CSF
HAT	Histone acetyltransferase
HDAC	Histone deacetylases
HDACi	HDAC inhibitor
HMGB1	High mobility group box 1
ICB	Immune checkpoint blockade
IDO	Indoleamine 2,3-dioxygenase
IGRT	Image-guided radiation therapy
IL-[number]	Interleukin-[number]
IMRT	Intensity-modulated radiation therapy
LCK	Lymphocyte-specific protein tyrosine kinase
LH	Luteinizing hormone
LHRH	Luteinizing hormone-releasing hormone
M-CSF	Macrophage CSF
M-MDSC	Monocytic MDSC
MDSC	Myeloid-derived suppressor cell
MMP	Matrix metalloproteinase
MSC	Mesenchymal stem cell
NAd	Noradrenaline
NDRG1	N-myc downstream-regulated gene
NEC	Neuroendocrine-like cell
NEPC	Neuroendocrine prostate cancer
NICE	National Institute for Health and Care Excellence
NK	Natural killer
NO	Nitric oxide
NOX2	NADPH oxidase 2
NRF2	Nuclear factor erythroid 2-related factor 2
PAMPs	Pathogen-associated molecular patterns
PAP	Prostatic acid phosphatase
PBMC	Peripheral blood mononuclear cell
PCa	Prostate cancer
PCNA	Proliferating cell nuclear antigen
PGE2	Prostaglandin E_2_
PIN	Prostatic intraepithelial neoplasia
PKA	Protein kinase A
PMN-MDSC	Polymorphonuclear MDSC
PNI	Perineural invasion
PSA	Prostate-specific antigen
RARP	Robot-assisted radical prostatectomy
RHAMM	Receptor for hyaluronan-mediated motility
RNS	Reactive nitrogen species
ROS	Reactive oxygen species
RP	Radical prostatectomy
RTK	Receptor tyrosine kinase
SAHA	Suberoylanilide hydroxamic acid
SCF	Stem cell factor
STAT3	Signal transducer and activator of transcription 3
TAM	Tumour-associated macrophage
TCR	T cell receptor
TGFβ	Transforming growth factor-beta
TI1IFN	Tumour intrinsic type 1 interferon
TIMP1	Tissue inhibitor of metalloproteinase 1
TLR9	Toll-like receptor 9
TME	Tumour microenvironment
TRAIL-R	TNF-related apoptosis-induced ligand receptor 2
Treg	T regulatory cell
TRUS	Transrectal ultrasound
UA	Uric acid
UPR	Unfolded protein response
VEGF	Vascular endothelial growth factor
Zol	Zoledronic acid
β-AR	β-adrenergic receptors
cAMP	Cyclic adenosine monophosphate
eNOS	Endothelial nitric oxide synthase
iNOS	Inducible nitric oxide synthase
mCRPC	Metastatic castration-resistant prostate cancer
mpMRI	Multiparametric magnetic resonance imaging
MSMB	Microseminoprotein-β
tNEPC	Treatment-dependent NEPC
